# A Questionnaire-Based Survey of Physical Medicine and Rehabilitation Residency Training in Pakistan

**DOI:** 10.7759/cureus.1005

**Published:** 2017-01-31

**Authors:** Farooq A Rathore, Aamir W Butt, Nabila Soomro, Noreen Akhtar

**Affiliations:** 1 Department of Rehabilitation Medicine, PNS Shifa Hospital, DHA II, Karachi 75500, Pakistan; 2 Department of Rehabilitation Medicine, Bahria University Medical and Dental College, Bahria University, DHA -II, Karachi; 3 Department of Rehabilitation Medicine, Faculty of Rehabilitation Medicine, University of Alberta, Edmonton, Canada; 4 Department of Rehabilitation Medicine, AFIRM, Rawalpindi, Pakistan; 5 Director, Institute of Physical Medicine and Rehabilitation, Dow University of Health Sciences (DUHS), Karachi, Pakistan; 6 Department of Rehabilitation Medicine, Combined Military Hospital, Abbottabad, Khyber Pakhtunkhwa, Pakistan

**Keywords:** physiatry, medical education, post graduate residents, post-graduate training, analysis, analysis, pakistan, south east asia, survey, developing country

## Abstract

Pakistan is one of the three countries in South East Asia that has an active postgraduate physical medicine and rehabilitation (PM&R) training program. College of Physicians and Surgeons Pakistan (CPSP) offers a four-year structured training program in PM&R. It consists of clinical teaching, lectures, rotations in other specialties, and writing a research dissertation.

The aim of this survey was to provide an objective analysis of the current PM&R training program, including the facilities available for training, the participation of residents in academic activities, and their participation in different PM&R procedures.

Hospital ethics committee approval was obtained. The questionnaire had sections on informed consent; basic demographics; the different components of residency training; and self-assessement of competence in different procedural skills. It was approved by the dean of PM&R at CPSP. There are six accredited training centers in Pakistan. Twelve residents are undergoing residency training at four different centers (Dec 2015). Key persons were nominated at each center to facilitate data collection. All residents (100% response rate) completed the survey. Almost all had read the CPSP training manual. Most had submitted the research dissertation. Training facilities varied across different centers, with the military center being the best equipped. The self-assessed competence of residents in different PM&R procedures varied among different centers, but overall it conformed to the competency levels specified in the training manual.

Overall PM&R residency training in Pakistan is satisfactory, but there is a need to strengthen the weak areas and standardize the training across all centers in the country.

## Introduction

Pakistan is one of the three countries in South East Asia that has an active postgraduate physical medicine and rehabilitation (PM&R) training program (the others being India and Bangladesh). College of Physicians and Surgeons Pakistan (CPSP) offers the only postgraduate specialist qualification in the subject of PM&R in the country [1]. It is a four-year structured training program carried out at accredited centers by CPSP-trained and approved supervisors. Physicians who have completed their house job (one-year internship) are eligible to take the Fellow of College of Physicians and Surgeons (FCPS) Part I exam in either surgery or medicine. Successful candidates can then join the FCPS Part II training in PM&R. Although Dr Kirmani and Dr Qazi laid the foundation for PM&R in Pakistan in the early 1960s, the formal FCPS training program in PM&R was started in 1998 by a group of pioneers who came together for the common goal of establishing a training program in the country [2]. The first fellow qualified the FCPS II exit exam in 2001; and so far, more than 55 physicians have passed the exit exams to become fellows, attending physicians, and faculty members. Most of them are serving in the Pakistan Army Medical Corps, while others are working in the public and private sector and a few are working in the Middle East.

There are six centers accredited for PM&R training inside the country [3], and one abroad at the Department of Physical Medicine and Rehabilitation, King Fahad Medical City, Riyadh, Kingdom of Saudi Arabia. Training facilities vary across different centers, and there is a need to standardize PM&R residency training in the country.

Measuring the quality of residency training programs is imperative to produce competent doctors and ensure patient safety [4]. Critical analyses and evaluations of residency training programs in different specialties have been published globally [5-8]. They help identify weaknesses, highlight strengths, and recommend future directions for their programs. We were unable to locate any published analysis of any residency program in Pakistan. The aim of this study is to provide an overview from the residents’ perspective of the current PM&R residency program in Pakistan, including the facilities available for training, the participation of residents in academic activities, and their participation in different PM&R procedures.

## Materials and methods

### Questionnaire development

The authors designed a self-administered questionnaire after mutual consultations and discussion. The authors are attending physicians in PM&R with professional experience ranging from 6–15 years. They are registered as faculty members in PM&R with the Pakistan Medical and Dental Council (PM&DC). Two of the authors (AWB and NS) were among the pioneer batches of residents who qualified FCPS-II in PM&R more than a decade ago. AWB, NS, and NA are current faculty members at CPSP and have been examiners and supervisors in PM&R for more than five years. NA is the only physiatrist in Pakistan with a degree in medical education. The lead author (FR) did clinical observerships in PM&R at leading institutes in the US, where he had a chance to closely observe the PM&R residency training program there and interact with the residents and faculty members. The questionnaire was sent for review and comments to the dean of the PM&R department at CPSP, who approved the content and format. The ethics review committee of the Armed Forces Institute of Rehabilitation Medicine (AFIRM), Rawalpindi, approved the research.

The questionnaire had four parts. The first part was informed consent, which explained the title and rationale of the study. It also assured the respondents of their anonymity, and that it would have no impact on their residency training in future or on their evaluations. The second part consisted of demographic data. It had questions about respondents' age, gender, institute, and year of residency training. The third part consisted of 21 questions. The respondents had to answer "Yes" or "No". The questions inquired if the resident had studied the PM&R training manual published by the CPSP; whether inpatient setup for training was available; whether regular clinical teaching rounds were carried out; and if training in electrodiagnostics was available in their department. It also asked about the residents' participation in national and international PM&R conferences, and if they had made posters or oral presentations. Residents were asked about their primary textbook for PM&R and their library or personal subscriptions to PM&R journals. In the end, residents were asked about their plans to go abroad for further training after completing the residency program.

The fourth part assessed the self-reported training level of residents in 11 different PM&R procedures. These included procedures required as core competencies in the training manual and some additional procedures. Residents had to mark themselves at one of the four levels: performed independently; performed under supervision; have only observed the procedure; and have not observed or performed the procedure. These are the levels of competency specified in the CPSP training manual.

At the end, space was provided for additional comments. Residents were thanked for their time and were again assured that responses will remain confidential and will not have any effect on their training and assessment.

### Data collection procedure

Data collection was done in Nov–Dec 2015. There are four centers with an active PM&R residency training program in Pakistan, with 12 residents undergoing training. The four residents enrolled in the FCPS training program at the Department of Physical Medicine and Rehabilitation, King Fahad Medical City, Riyadh, Kingdom of Saudi Arabia, were excluded from this survey. A supervisor or a key resource person at the institute was approached for the data collection. Each was briefed in advance and was provided written guidelines to facilitate the data collection (see Appendix). All residents gave consent to participate in the survey. The residents at each center were provided a blank copy of the questionnaire in an unmarked envelope. The questionnaire response time was estimated to be approximately 10 minutes. Therefore, respondents were requested to fill in the questionnaires on the spot. The email address and phone number of the corresponding author was provided for questions and clarifications. The residents completed the questionnaire, placed it in the unmarked envelopes, sealed it, and handed it over to the key resource person. Response rate was 100%. No financial or other compensation was offered for completing the survey.

### Data analysis

Data were analyzed using Statistical Package for the Social Sciences (SPSS) V 20 (IBM, New York). Descriptive statistics were used to compute the frequencies of basic demographics (age, location of institute) and different responses while mean and standard deviation (SD) was calculated for age.

## Results

There were 12 residents from four centers, totalling 100% of the resident population (Institute of Physical Medice and Rehabilitation (IPM&R), Karachi; Mayo Hospital, Lahore; Armed Forces Institute of Rehabilitation Medicine (AFIRM), Rawalpindi; and Combined Military Hospital (CMH), Peshawar). Two centers had no supervisor nor resident in PM&R (The Children’s Hospital and the Institute of Child Health, Lahore, and Combined Military Hospital (CMH), Muzaffarabad, Azad Kashmir). Most of the residents were female (n=9), married (n=10), in the fourth year of their training (n=6), and were from the Army Medical Corps (n=7).The mean age was 30.6 + 2.9 years (range 26–36 years) (Table 1).

**Table 1:** Demographics of the respondents

Residents in rehabilitation medicine (country-wide statistics)	Total: 12
Armed Forces Institute of Rehabilitation Medicine	5
Combined Military Hospital, Peshawar	2
Institute of Physical Medice and Rehabilitation, Karachi	3
Mayo Hospital, Lahore	2
Gender distribution	
Male	3
Female	9
Age (years)	
25–30	6
31–35	5
35–40	1
Year of residency	
1st year	2
2nd year	3
3rd year	1
4th year	6
Marital status	
Married	10
Single	2
Topic of the dissertation thesis	
Have not submitted the synopsis	3
Musculoskeletal rehab	4
Neurological rehab	2
Outcome measures	1
Pain management	1
Quality of life	1

 Almost all (n=11) had read the PM&R training manual of CPSP. Regular lectures are delivered at most centers, and there are regular assessments of trainees. The majority (n=10) have received training in medical writing and have attended national PM&R conferences; but only one (fourth-year resident) has published a manuscript. Institutional access to PM&R journals was not available to any resident, and none of them had a current personal subscription to any PM&R journal. DeLisa's *Physical Medicine and Rehabilitation: Principles and Practice* was the most widely used textbook by all residents, although other resources and books were also regularly consulted. Almost all (n=10) have plans to go abroad for further training/observerships/fellowships (Table 2).

**Table 2:** Detailed responses of the residents PM&R: physical medicine and rehabilitation CPSP: College of Physicians and Surgeons Pakistan FCPS: Fellow of College of Physicians and Surgeons

Questions	Yes	No
Have you read the manual of training for PM&R published by CPSP?	11	1
Do you have regular lectures on topics related to your specialty by the faculty? ( weekly, fortnightly, monthly)	9	3
Do you have inpatient rehabilitation for the training of residents?	7	5
Are teaching ward rounds carried out in your department?	2	10
Is journal club regularly held at your department/hospital/institute?	10	2
Is training in electrodiagnostics (electromyography/nerve conduction story) available at your department? If not, where do you go for training?	7	5
Is there a system of regular and periodic assessment of residents (monthly/quarterly exams) in your department/hospital/institute?	10	2
Are you currently or in the past have been involved in medical writing? (Does not include CPSP synopsis and dissertation writing.)	5	7
Have you published a manuscript (in any category)?	1	11
Have you received any formal training in medical research and writing at your department/hospital? (Excludes CPSP workshops.)	10	2
Have you attended a PM&R conference?	10	2
Have you delivered an oral/platform presentation in a medical conference/symposium?	2	10
Have you done a poster presentation in a medical conference/symposium?	5	7
Are you member of a professional medical society? Please specify: National International	12	-
Do you have a departmental/institutional library?	10	2
Do you have institutional access to rehabilitation medicine journals (via the library)?	0	12
Do you have a personal/society subscription to a rehabilitation medical journal?	0	12
Do you perform on-call duties?	6	6
Has your dissertation synopsis been accepted?	7	5
After qualifying FCPS, do you have plans of going abroad for further training/observership/fellowship?	10	2

Residents' self-assessed competence in different PM&R procedures varied between different centers, but overall it conformed to the competence levels specified in the training manual. Most of the residents had independently passed an indwelling catheter (n=10), done manual muscle testing (n=10), had managed autonomic dysreflexia (n=7), and had performed an intra-articular injection of the knee or shoulder for pain management (n=7). Three residents had observed the procedure of urodynamics and the rest (n=9) had not. Only one fourth year resident had done independent electrodiagnostic studies while three had done so under supervision. (All of them were from a single center: AFIRM) (Table 3).

**Table 3:** Self-assessed competence of residents in PM&R procedures

Procedure	Level of competence			
	Performed independently	Performed under supervision	Have only observed the procedure	Have not observed or performed the procedure
Nerve conduction studies/electromyography	1	3	6	2
Urodynamics	-	-	3	9
Foley catherization	10	1		1
Clean intermittent catheterization	3	3	3	3
Management of autonomic dysreflexia	7	2	-	3
Manual muscle testing (muscle power charting)	10	-	1	1
Intra-articular injections (knee and shoulder)	7	1	4	-
Fluoroscopic guided spinal injections	-	1	4	7
Phenol blocks for spasticity management	1	3	4	4
Botulinum toxin injection for spasticity management	-	2	8	2
Pain management procedures (trigger point injections, carpal tunnel syndrome injection)	4	2	6	-

## Discussion

This is the first survey from Pakistan that analyzes PM&R training programs. It included all residents currently enrolled in the FCPS residency training program in Pakistan. Although the number of residents enrolled in the various CPSP fellowship programs (Nov 2015) is more than 21,276 [9], only 12 of these are from PM&R. In addition, two training centers are dormant. This should be a matter of concern, and steps should be implemented to increase the number of PM&R residents in future in order to combat the rising burden of disability in Pakistan. One of the reasons for this low number of PM&R residents might be lack of exposure regarding PM&R among medical students [2].

The survey revealed wide variations in the residency programs at different centers. Some centers are well-equipped and have all the elements of residency training (including lectures, teaching rounds, adequate infrastructure, and regular assessment), while other centers lack these facilities. Although most of the residents had read the CPSP training manual for PM&R, the manual itself was written in 2002. It was recently revised in 2016, by incorporating the recent advances in PM&R. Now there is a need to implement all the elements of training mentioned in the manual in the residency training programs across the country.

Inpatient rehabilitation is an essential element of training for residents. This facility is available only at two military centers (AFIRM, Rawalpindi, and CMH, Peshawar). Training based only on outpatient consultations and attending calls from other specialties lacks critical components in teaching the essential principles of PM&R. This needs to be addressed in order to improve the training.

Electrodiagnostic training facilities are available only at the military training centers, and the residents from the other two centers visit these military centers for training in electrodiagnostics. A system of periodic assessment by monthly or quarterly exams can assess if the residents are learning the required skills and advancing their knowledge. Assessments in the form of written exams, viva voice, and task-oriented assessments of clinical skills are regularly done at three centers.

Journal club meetings form an important component of residency training in any specialty. They encourage regular consultation of the literature and can result in enhanced medical knowledge, improved patient care, better understanding of biostatistics and research competency, and better critical literature appraisal skills [10-12]. This activity is regularly conducted at most PM&R training centers, although the format and content varies among centers. Mostly, it is limited to selecting and presenting a recent review article on a topic and then discussing its relevance and application to local circumstances. There is a need to revise the current format to include original research articles and incorporate the principles outlined by Deenadayalan, et al. [13].

The residents do not have institutional access to PM&R journals. This problem is not specific to PM&R, but probably reflects the lack of well-established medical library services in the majority of institutes and hospitals in Pakistan.

Medical research and writing is rapidly progressing in Pakistan [14]. There has been a paradigm shift in the research output of Pakistani physiatrists in the last decade, and residents and young fellows are spearheading much of this change. This is also evident from this survey. Most of the residents had received training in medical research and writing, mostly in the form of lectures and workshops on research writing. This training was in addition to the mandatory workshops by CPSP on communication skills, research writing, biostatistics, and internet skills [15]. This was not the case a decade ago. Many current residents have had their mandatory dissertation synopsis approved, and some have already completed their dissertation writing. Residents' involvement in research and writing from the start equips them with the essential skills that will help them survive in a competitive academic environment marked by the pressure to publish or perish. PM&R residents have already been making their mark: the residents from AFIRM have been participating in the annual Research Neurology Day at Shifa College of Medicine, Islamabad, since 2007; they have won many grants and other prizes for best posters and oral presentations.

Due to the large volume of patients they see, the residents are exposed to a diverse range of patients with different diseases. This is a strength of the current training system, and it allows residents to examine a large number of patients and see nearly all the diseases mentioned in textbooks. FR noticed during his stay in the US that the annual average number of patients with stroke, spinal cord injury (SCI), pediatric disabilities (particularly cerebral palsy and myopathies), and rheumatologic disabilities seen by a Pakistani PM&R resident was much higher than the number of patients seen by a resident working in the US. This might be a reason why almost all the residents surveyed appeared to have achieved the procedural competencies outlined in the training manual.

Many physiatrists who have qualified from the current PM&R residency program in Pakistan have travelled abroad for clinical observerships and fellowships. Some of them are working as attending physicians in the Middle East. This is a testimonial to the rigorous quality of the current residency program in Pakistan.

Some major revisions are underway to improve the current PM&R residency training program in Pakistan. These include revision of the training manual, recruitment of new faculty members, faculty development workshops, improving the examination structure, and introducing an intermediate module at two years of training. It is hoped that these changes will lead to a better and stronger PM&R residency training program that will be able to produce physiatrists better equipped to face the growing burden of disability in Pakistan.

### Strengths and limitations

This is the first survey to present an objective analysis of the PM&R residency training program in Pakistan. We successfully gathered data from all residents based in Pakistan. The survey has comprehensively addressed the different aspects of the residency training program being carried out by CPSP.

The survey did not include the opinions and feedback of the course directors and supervisors. We did not formally assess residents' competency level in procedural skills or knowledge domains. In addition, comparisons with similar residency programs in other subjects or with PM&R residency programs in other countries was not made.

### Recommendations for the future

The PM&R residency program in Pakistan is structured, competitive, and offers many opportunities to the willing resident to learn. However, there is a need to standardize the training across the country. Moreover, there is a need to develop subspecialty programs in PM&R, including spinal cord injury medicine, pediatric rehabilitation, neurorehabilitation, cancer rehabilitation, cardiac rehabilitation, etc.

Plans are already underway to conduct a similar and more comprehensive survey in Bangladesh and India to assess and compare PM&R residency programs across countries in the same region. In addition, a SWOT analysis of the current PM&R training program will be conducted by getting structured feedback from all stakeholders (residents, supervisors, examiners, and faculty members). PM&R training varies across different countries, with each system having its unique strengths and peculiar weaknesses. Therefore, there is a need for closer collaboration and coordination between different national examining and accreditation bodies, the International Society of Physical and Rehabilitation Medicine (ISPRM), different regional societies (e.g., European Society of Physical and Rehabilitation Medicine (ESPRM), Asia-Oceania Society of Physical and Rehabilitation Medicine (AOSPRM)), and subspecialty societies so that a curriculum and minimal competency levels of training can be defined for PM&R training across the globe.

## Conclusions

The residency training program in PM&R in Pakistan is relatively young but has substantially improved in the last decade. This model can be successfully implemented in similar low resource areas to facilitate the development of academic PM&R. There is a need for continuing improvement and the implementation of the current curriculum uniformly across all training centers in order to produce better-qualified physiatrists.

The developed regions should facilitate the transfer of PM&R knowledge to low resource countries and promote residents and faculty exchange programs in order to develop PM&R in the developing world.

## Appendices

### Instructions and suggestions for filling data forms for the research project titled "Analysis of the Rehabilitation Medicine Residency Program in Pakistan"

Plan ahead to save time and energy.

Ensure that you have an adequate number of blank questionnaires as per the estimated number of trainees in the institute.

Please call all trainees and assure them of the anonymity of the questionnaire, and that this will have no effect on their training, assessment, and examinations.

Nominate a senior trainee (resident) as the coordinator, and make him/her responsible for distributing and collecting the completed questionnaires.

Hand over the blank questionnaires enclosed in marked envelopes to the residents. Ask them to fill in the questionnaire immediately. In case of any queries or clarifications, they can contact Dr Farooq Rathore ( 0312-9549821).

Explain the rationale of the research project and assure the trainees that it will not take more than 10 minutes for the whole exercise.

Ask the trainees to fill in the questionnaire right there to the best of their knowledge. Instruct the trainees to carefully study and answer the questions, as cutting would render that particular answer null, and it would be excluded from the final analysis. Ask the trainees to circle the correct answer.

Once the questionnaire has been filled, put it in the marked envelope and seal it. Hand over the envelope to the senior trainee who will deposit it with the senior consultant or mail it directly to the address below.

Dr Farooq Rathore, MBBS, FCPS, OJT (USA)

Assistant Professor, Department of Rehabilitation Medicine,

Combined Military Hospital and CMH Lahore Medical College, 

Abdul Rahman Road, Lahore Cantt, 54810.

Once the questionnaires have been posted, please send a SMS on 0312-xxxxxx or email

Thank you very much for your cooperation and time.
